# Long COVID-19 olfactory dysfunction: discrepancy between psychophysical tests and self-perception

**DOI:** 10.1016/j.bjorl.2026.101797

**Published:** 2026-03-09

**Authors:** Aina Sansa, Alda Cardesín, Mariana Campos, Carlota Rovira, Josep de Haro, Elios Yuste, Miguel Caballero-Borrego

**Affiliations:** a Hospital Parc Taulí, Otorhinolaryngology Department, Rhinology and Sleep Unit, Sabadell, Spain; b Hospital Municipal Badalona, Otorhinolaryngology Department, Badalona, Spain; c Grupo Investigación Interdisciplinar de Asesoramiento de la Percepción (GIIASP), Spain; d Universitat de Barcelona, Faculty of Medicine and Health Sciences, Barcelona, Spain; e Otorhinolaryngology Department, Hospital Clinic de Barcelona, Barcelona, Spain; f Institut d'Investigacions Biomèdiques Agusti Pi Sunyer (IDIBAPS), Spain

**Keywords:** Quality of life, COVID-19, Olfactory dysfunction, BAST-24, Rhinitis

## Abstract

**Objectives:**

To evaluate the discrepancies between psychophysical olfactory tests and self-perceived smell function in consecutive patients with long COVID-19-related olfactory dysfunction, and to compare the characteristics of this impairment with those observed in other nasal conditions, in order to improve diagnostic accuracy and guide targeted treatment strategies.

**Methods:**

An observational study of 86 long COVID-19 patients assessed by the Barcelona Smell Test (BAST-24 Plus), Visual Analogue Scale (VAS), and the quality-of-life Sino-Nasal Outcome Test (SNOT-22). Results were compared with 120 healthy controls and 121 patients with chronic rhinosinusitis with Nasal Polyps (CRSwNP).

**Results:**

Long COVID-19 patients reported a mean VAS score of 6.34 and a SNOT-22 score of 26.43, with question 21 (smell) accounting for 12.67% of the total. No correlation was found between VAS and total SNOT-22 score, but a moderate positive correlation was observed with question 21 (*r* = 0.488, p < 0.001). BAST-24 Plus scores were significantly lower than in controls for Detection, Identification, and Correct Answer (p < 0.001). Compared to the CRSwNP group, long COVID-19 patients showed less impairment in Detection and Identification (p < 0.001). A stronger negative correlation was observed between VAS and Identification (*r* = −0.629, p < 0.0001) than with Detection (*r* = −0.482, p < 0.0001), suggesting that odor identification dysfunction has a greater impact on subjective perception.

**Conclusions:**

The subjective perception of olfactory dysfunction does not correlate with psychophysical test results in long COVID-19 patients and is characterized by a predominantly identification-dominant quantitative impairment, which differs from other disorders showing detection-dominant impairment. These findings highlight the need for specific diagnostic and therapeutic strategies.

**Level of evidence:**

III.

## Highlights

• Many COVID-19 patients experience olfactory dysfunction.
• Self-reported olfactory complaints often do not match the results of smell tests.
• Long COVID shows a predominantly identification-dominant olfactory impairment.
• Recognizing key features helps refine diagnosis and guide targeted treatment.

## Introduction

Infection with SARS-CoV-2, the virus responsible for COVID-19, has been one of the most significant pandemics in recent history, causing a wide range of clinical manifestations. Although the clinical severity of COVID-19 has decreased over time, a considerable number of patients continue to experience persistent symptoms and require regular follow-up with otolaryngology specialists.^1^ Among the most prevalent of these symptoms is olfactory dysfunction, which, although initially underestimated, has gained increasing clinical and scientific attention. Recent studies have demonstrated that olfactory dysfunction can occur in a significant proportion of patients, even in the absence of other respiratory symptoms, and may persist for an extended period,^2^ potentially serving as a marker for long-term sequelae of the disease.^3^, ^4^, ^5^

The prevalence and duration of olfactory dysfunction have been shown to vary according to the predominant SARS-CoV-2 strain in each phase of the pandemic.^6^ While early variants such as Alpha and Beta were associated with high rates of anosmia and hyposmia, with some studies reporting prevalence rates of up to 68%,^7^ the emergence of the Omicron variant led to a significant reduction in the incidence of olfactory dysfunction, with rates dropping below 20% in certain cohorts. However, the risk of long-term symptoms has not been entirely eliminated.^8^^,^^9^ This variability suggests that the underlying pathogenic mechanisms may differ between viral strains, and that the evolution of the virus has influenced the prevalence and severity of olfactory dysfunction.

Olfactory dysfunction is a common feature of various conditions involving generalized inflammation of the nasal mucosa, as seen in many respiratory infections and in chronic rhinosinusitis. In COVID-19, olfactory dysfunction has occasionally been observed in the absence of other respiratory symptoms, suggesting a distinct pathophysiological mechanism beyond the non-specific inflammatory damage to the nasal mucosa.^9^ It has been proposed that SARS-CoV-2 may directly affect the sustentacular cells of the olfactory epithelium, resulting in neurosensory dysfunction.^10^^,^^11^ These different pathophysiological mechanisms underscore the need for a more detailed analysis of the characteristics of olfactory dysfunction to develop effective, tailored treatment.

Several tools are available to assess olfactory dysfunction. Psychophysical and instrumental tests enable both quantitative and qualitative evaluations of the patient's actual olfactory capacity. In addition, tools such as the Visual Analog Scale (VAS) can be used to assess the patient's subjective perception of their condition, though this may not always accurately reflect the true extent of olfactory dysfunction.^12^ VAS scores, while useful for indicating the severity of olfactory dysfunction, may be influenced by the patient's adaptation to the condition and their awareness of the loss of smell.^13^

The main aim of this study is to analyze whether olfactory dysfunction in patients with long COVID-19 presents specific features that distinguish it from olfactory dysfunction caused by other rhinological conditions, such as CRSwNP. Despite widespread use of patient-reported measures, subjective smell loss often diverges from psychophysical performance, potentially leading to misclassification and suboptimal counselling. Long COVID offers a unique model to study this discrepancy using validated tools. Our study explicitly compares long COVID with CRSwNP with the same psychophysical battery, highlighting where patient perception aligns ‒ or not ‒ with specific olfactory domains (Detection vs Identification), and the clinical consequences of such mismatches.

Secondary aims are to investigate whether there is a discrepancy between the subjective perception of olfactory dysfunction and psychophysical test results, and to assess the impact of olfactory dysfunction on quality of life in patients with long-term COVID-19. A better understanding of the characteristics of olfactory dysfunction in these patients may allow for the development of targeted treatments that differ from those used for other rhinological disorders.

## Methods

### Population

Prospective observational study conducted between 2022 and 2024 at the Rhinology Unit of a tertiary care hospital. A total of 86 patients were included if they met the following inclusion criteria: age ≥18-years, a confirmed history of SARS-CoV-2 infection (via diagnostic testing during the acute phase or through subsequent serology), and persistent olfactory dysfunction lasting more than six months following the acute phase of the disease. Exclusion criteria included the presence of concomitant structural or inflammatory conditions that could account for the olfactory dysfunction, as determined through medical history and nasal endoscopic examination performed by an otolaryngologist.

The study and all procedures were conducted in accordance with the ethical standards of the 1975 Declaration of Helsinki, as revised in 2008, and the national and institutional guidelines on human research. The study protocol was approved by the Ethics Committee of our institution (reference: 2022/5011). Participation was entirely voluntary, and no compensation was provided to subjects for their involvement. Written informed consent was obtained from all subjects, and the anonymity was ensured throughout the study.

### Control groups

CRSwNP patients and healthy controls were prospectively enrolled. Each participant completed a standardized medical history, nasal endoscopic examination, and BAST-24 assessment under our routine clinical protocol. The inclusion and exclusion criteria were:

• Healthy controls: Adults without current/past chronic rhinosinusitis, nasal surgery, or neurodegenerative disease; VAS = 0; and normal endoscopy. Exclusion criteria: recent upper respiratory infection (< 4-weeks), pregnancy, and any condition affecting olfaction.
• CRSwNP: Consecutive adults with CRSwNP grades I–II (Lildholdt) confirmed by endoscopy. Exclusion criteria: recent systemic corticosteroids (< 8-weeks), acute exacerbation/infection within a similar window, prior smell training, prior skull-base surgery, or neurodegenerative disease.

To reduce confounding, we included only patients with early-stage CRSwNP (grades I–II), and those who had received prior corticosteroid treatment were excluded from the study.

### Variables

Demographic data collected included age (analyzed in predefined age groups), sex (male or female), and smoking status (non-smoker, smoker, or ex-smoker).

All patients with confirmed COVID-19 underwent the Barcelona Smell Test-24 (BAST-24 Plus),^14^ the Visual Analogue Scale (VAS),^15^ and the Sino-Nasal Outcome Test-22 (SNOT-22).^16^^,^^17^ All questionnaires have been validated for the studied population. Psychophysical testing and questionnaire administration were conducted by nursing staff from the Rhinology Unit, specifically instructed in olfactory assessment.

The BAST-24 Plus is a psychophysical olfactory test that evaluates 20 odors linked to the olfactory nerve (cranial nerve I) and 4 odors associated with the trigeminal nerve (cranial nerve V). The test evaluates three main aspects of smell: (1) Odor detection (patients indicate whether they perceive each of the 24 presented odors); (2) Spontaneous identification (patients attempt to name each odor without options); and (3) Correct answer in forced choice (patients select the correct odor from four multiple-choice options). Results from patients in the COVID-19 group were compared with those from a healthy control group of similar ages and characteristics, as well as with patients diagnosed with CRSwNP grade I and II (according to the Lildholdt scale).^18^

The Visual Analog Scale (VAS) was used to assess an individual's self-perception of olfactory function. Scores range from 0 (normal smell perception) to 10 (complete loss of smell). A VAS score of 0 was required for inclusion in the healthy control group.

The Sino-Nasal Outcome Test-22 (SNOT-22) is a validated instrument used to evaluate disease-specific quality of life in patients with sinonasal conditions. It includes 22 questions covering nasal, otologic, facial, general, psychological symptoms, and sleep-related symptoms. Each question is scored from 0 (no problem) to 5 (severe problem). Total scores range from 0 to 110, with higher scores indicating worse quality of life. In this study, both the total score and item 21 ‒ which specifically address the impact of smell and/or taste loss on quality of life ‒ were analyzed separately.

All variables related to olfactory dysfunction referred to quantitative impairment, including detection, identification, and correct answer scores of BAST-test. Qualitative disorders (e.g., parosmia, phantosmia) were not assessed in this study.

### Statistical analysis

The sample size was calculated to achieve a statistical power of 80% with a two-sided alpha error of 5% to detect a ≥20-point absolute difference in BAST-24 Detection between CRSwNP and long COVID, assuming ≈70% Detection in CRSwNP based on pilot observations. This yielded minimums of 56 long COVID and 111 CRSwNP participants for two-sided testing with continuity correction. Our final sample exceeded these thresholds (86 and 121, respectively).

Patient characteristics are reported as medians, means, and Standard Deviation (SD) for quantitative variables, and as absolute frequencies and percentages for qualitative variables. Continuous variables were compared using Student's *t*-test when the assumptions of normality and homogeneity of variances were met, and the Wilcoxon-Mann–Whitney test otherwise. Qualitative variables were compared using the Chi-Squared or Fisher's exact test. Correlation between two quantitative variables was assessed using Pearson's correlation when the assumptions of normality and homoscedasticity were satisfied. Otherwise, Spearman's rank correlation coefficient was applied. All statistical analyses were performed using R software (version 4.0.2; R Foundation for Statistical Computing, Vienna, Austria). A p-value of <0.05 was considered statistically significant.

## Results

A total of 86 patients with long COVID-19 olfactory dysfunction were included, with symptom duration ranging from six months to four years.

The long COVID-19 group had a mean age of 49.07 years (range: 18–83 years), with a predominance in the 51–70 age range (43.02%). The majority of participants were female (76.7%) and non-smokers (62.8%) (Table 1).

**Table 1:** Demographic characteristics of the long COVID-19 population.

			p
Mean age, years (standard deviation)		49.07 (14.19)	
Age (years)	18‒30	10 (11.63%))	<0.001^a^
	31‒50	34 (39.53%	
	51‒70	37 (43.02%)	
	> 70	5 (5.81%)	
Sex	Male	20 (23.3%)	<0.001^a^
	Female	46 (76.7%)	
Smoking history	Non-smokers	54 (62.8%)	<0.001^a^
	Smokers	20 (23.3%)	
	Ex-smokers	12 (13.9%)	

a A p-value < 0.05 is considered statistically significant.

Long COVID-19 patients had a mean total SNOT-22 score of 26.43. Item 21 of the SNOT-22, which specifically assesses the impact of olfactory dysfunction on quality of life, had a mean score of 3.35, indicating a substantial impact of this specific question on the overall test score. The mean score on the Visual Analog Scale (VAS) was 6.34, whereas, by definition, the healthy control group had a VAS score of 0 (Table 2).

**Table 2:** Comparison between the long COVID-19 population and the Healthy control group.

	COVID-19	Control	p-value
	Mean (SD)	Mean (SD)	
Age (years)	49.07 (14.19)	42.0 (1.70)	<0.001^a^
SNOT-22 (total)	26.43 (20.0)	‒	
SNOT-22 (Question 21)	3.35 (1.22)	‒	
Visual Analogue Scale (VAS)	6.34 (2.26)	0.0	
BAST-24: odor Detection	90.81 (22.04)	99.44 (1.70)	<0.001^a^
BAST-24: odor Identification	52.15 (25.44)	65.35 (18.14)	<0.001^a^
BAST-24: Correct Answer forced choice	34.94 (20.42)	74.13 (11.76)	<0.001^a^

a A p-value <0.05 is considered statistically significant.

In the psychophysical assessment using the BAST-24 Plus test, the long COVID-19 group showed the following mean scores: 90.81 in Odor Detection, 52.15 in Identification, and 34.94 in Correct Answer. The results obtained with the BAST-24 Plus in patients with long COVID-19 were compared with 120 subjects in the healthy control group. Long COVID-19 patients showed significantly lower BAST-24 Plus scores (p < 0.001) compared with healthy controls in all three domains: Detection, Identification, and Correct Answer (Table 2).

The patient's self-perception of olfactory dysfunction (VAS-values) did not correlate with overall quality of life (SNOT-22) (correlation coefficient 0.067, p = 0.540). However, when VAS scores were specifically compared with item 21 of the SNOT-22 (impact of smell on quality of life), a moderate positive correlation was observed (correlation coefficient: 0.488, p < 0.001).

**Fig. 1:**
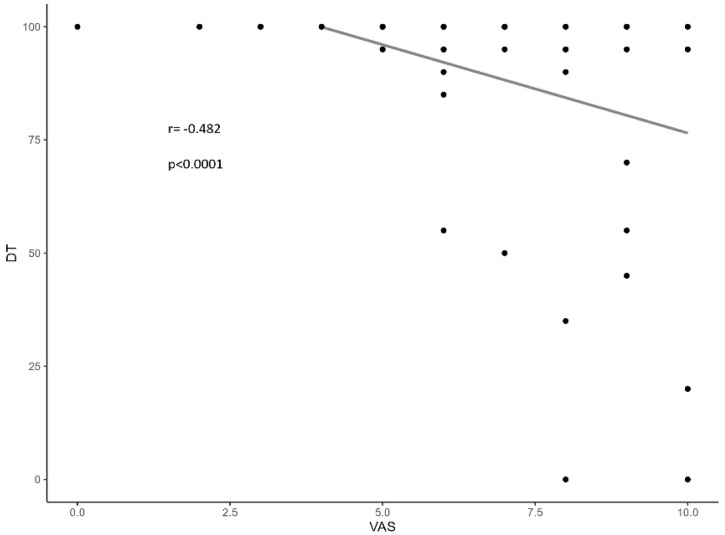
Dot plot illustrates the relationship between Visual Analogue Scale (VAS) and Odor Detection (DT) in COVID-19 group, showing a moderate negative correlation (*r* = −0.482) (p < 0.0001).

**Fig. 2:**
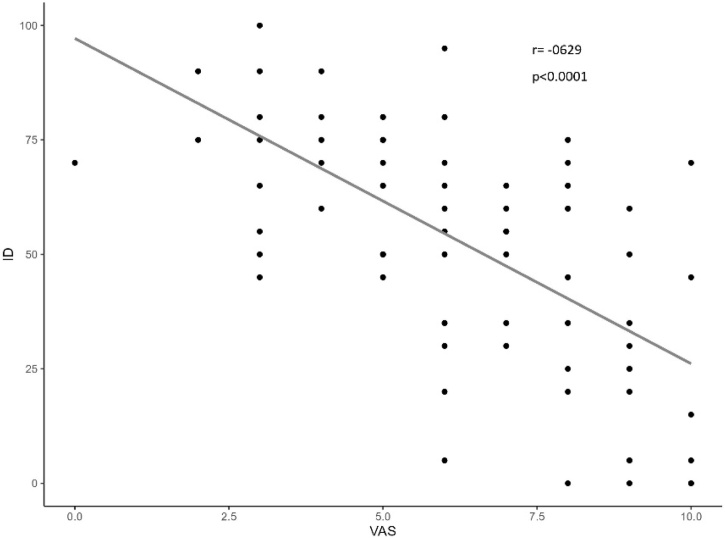
Dot plot illustrates the relationship between Visual Analogue Scale (VAS) and Odor Identification (ID) in COVID-19 group, showing a moderate negative correlation (*r* = −0.629) (p < 0.0001).

Dot plots were used to compare the subjective perception of olfactory dysfunction (VAS) with the actual olfactory function, as assessed by psychophysical testing (BAST-24 Plus). In the long COVID-19 cohort, the inability to Identify odors had a greater significant negative impact on VAS scores than did simple odor Detection. The VAS showed a moderate negative correlation with Detection (*r* = -0.482, p < 0.0001) (Fig. 1), while the correlation was stronger with Identification (*r* = -0.629, p < 0.0001) (Fig. 2).

**Fig. 3:**
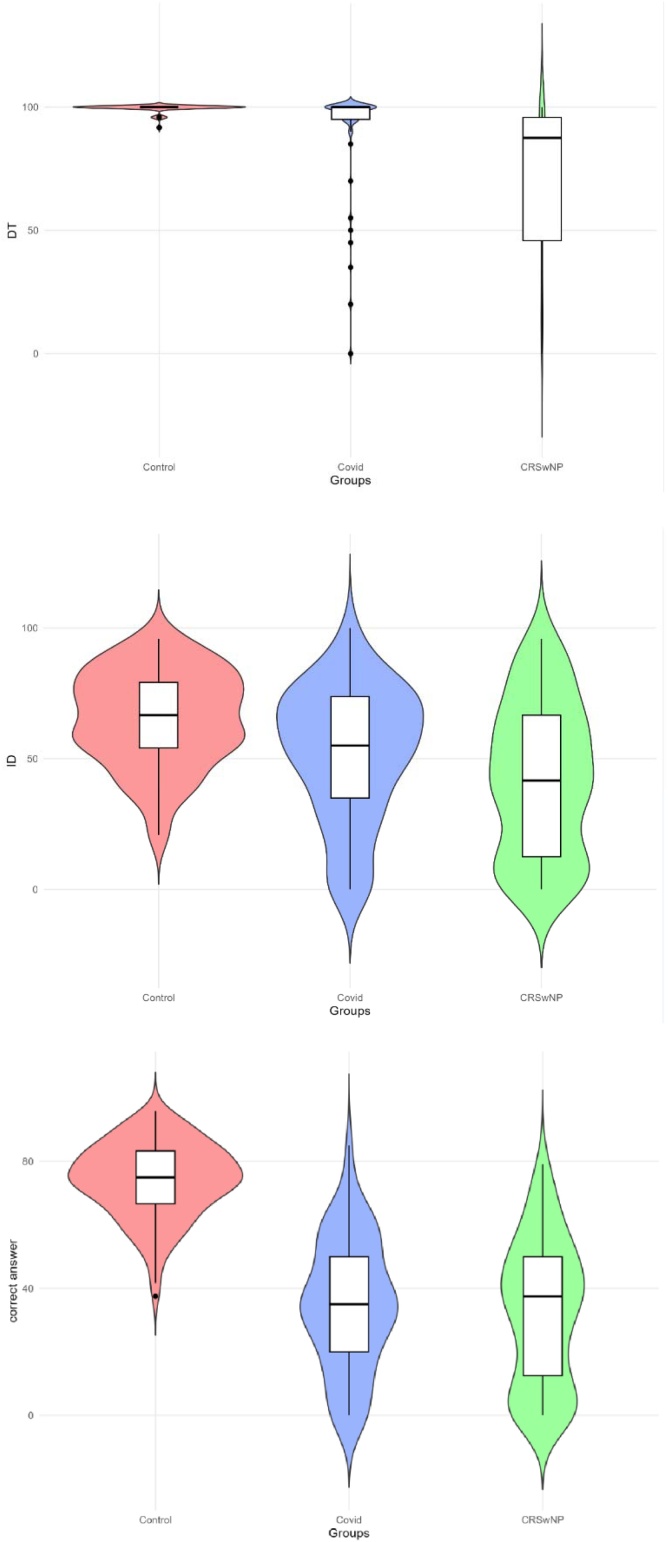
Density distribution plots of BAST-24 Plus scores in the three domains: Odor Detection (top), Identification (middle), and Correct Answer (bottom), across healthy controls, long COVID-19 and chronic rhinosinusitis with polyps groups (CRSwNP).

The different variables of the BAST-24 Plus were also compared between the long COVID-19 group and a cohort of 121 patients with CRSwNP, matched by mean age and standard deviation (Table 3). Patients with CRSwNP showed more severe olfactory dysfunction, with lower scores in all three domains of the BAST-24 Plus, although the differences were statistically significant for Odor Detection and Identification, but not for Correct Answer (Fig. 3).

**Table 3:** Comparison between the long COVID-19 population and the population with Chronic Rhinosinusitis with Nasal Polyps (CRSwNP).

	COVID-19	RSCwNP	p-value
	Mean (SD)	Mean (SD)	
Age (years)	49.07 (14.19)	47.14 (16.17)	0.364
BAST-24: odor Detection	90.81 (22.04)	72.07 (32.67)	<0.001^a^
BAST-24: odor Identification	52.15 (25.44)	42.42 (28.88)	<0.001^a^
BAST-24: Correct Answer forced choice	34.94 (20.43)	32.13 (22.59)	0.414

a A p-value < 0.05 is considered statistically significant.

## Discussion

Despite the variability in olfactory dysfunction associated with different predominant SARS-CoV-2 variants, this condition has emerged as a relevant clinical marker that persists in a subset of patients over the long term.^19^ Moreover, its characteristics differ from those observed in other chronic infections, highlighting the need for specific diagnostic strategies that assess the various dimensions of olfaction, as well as appropriate follow-up and rehabilitation approaches.^2^^,^^20^

Regarding self-perceived olfactory dysfunction, most long COVID-19 patients reported consistently high scores in the VAS scale, consistent with findings reported by others.^21^ In terms of quality of life, item 21 of the SNOT-22 questionnaire, which focuses on olfactory dysfunction, was the most affected, whereas the overall score of the questionnaire was less severely impacted. A positive correlation was found between the VAS and item 21 of the SNOT-22, but not with the total score of the questionnaire, suggesting that the overall SNOT-22 score in this population is primarily influenced by the negative impact of olfactory dysfunction on quality of life. Other studies have reported a significant association between the SNOT-22 total score and patient-reported olfactory loss,^22^ but the specific impact of item 21 remains unclear, as it was not analyzed separately in those studies, as was done in ours.

In the psychophysical assessment using the BAST-24 Plus, long COVID-19 patients showed significantly lower scores in Detection, Identification, and Correct Answer compared to healthy controls, as expected. However, Detection scores remained very high (>90 out of 100), despite patients reporting a high perception of olfactory dysfunction (mean VAS-score of 6.34). This supports the notion that subjective perception of olfactory disfunction does not always correlate with the actual degree of dysfunction, as other authors have previously suggested.^12^ In our long COVID-19 cohort, the factor most strongly associated with a worse VAS score was olfactory Identification, rather than Detection. This highlights that the inability to identify odors has a greater negative impact on subjective perception (VAS-score) than merely detecting them, a finding also reported by other authors.^23^ Therefore, according to our study, the quality of life in these patients is more significantly affected by Identification olfactory dysfunction than by Detection impairment. For this reason, it is crucial to conduct a comprehensive olfactory assessment using psychophysical tests that evaluate multiple dimensions of olfaction, rather than solely focusing on detection. This approach will allow for the design of more appropriate treatments for these patients.

Olfactory dysfunction may arise from diverse etiologies, with different underlying pathophysiological mechanisms and patient impacts. When comparing our findings in the long COVID-19 patients with the study by Alobid et al.^24^ on patients with CRSwNP, we observe that the VAS scores were similar, although BAST-24 Plus scores were worse in the CRSwNP group. This suggests that the VAS may not strictly reflect the degree of olfactory dysfunction, but rather the patient's perceived burden of smell loss, which may vary depending on the underlying condition and the pathophysiological mechanisms of each disorder. In COVID-19, olfactory dysfunction appears to be related to direct damage to the olfactory epithelium and sensorineural dysfunction,^25^ as in others viral infections,^21^ whereas in CRSwNP, inflammation and mechanical obstruction of the nasal mucosa play a major role.^26^ These pathophysiological differences have direct implications for the interpretation of olfactory test results and the selection of specific therapeutic strategies for each condition. Some authors have correlated the grade of olfactory dysfunction with the severity of the CRSwNP.^27^ In our CRSwNP population, despite being in the early stages, olfactory impairment was more pronounced than in the long COVID-19 population. Comparing BAST-24 scores between the severe CRSwNP group from Alobid et al.'s study and our early-stage CRSwNP population, as expected, Detection was more impaired in the severe CRSwNP group (57.4 vs. 72.1). However, the mean scores for Identification (42.1 vs. 42.4) and Correct Answer in forced choice (37.9 vs. 32.1) were similar in both populations, despite comparison between advanced and early stages.^24^

The analysis of all these data reinforces the need to use both psychophysical tests and subjective scales for a comprehensive assessment of olfactory dysfunction, as they provide complementary information.

### Clinical translation

In practice, a patient with near-normal Detection but low Identification may still experience substantial perceived disability. Rehabilitation should therefore emphasize identification-focused smell training and cognitive-perceptual strategies, while Detection alone may underestimate impact. Compared with CRSwNP ‒ where mucosal inflammation/obstruction drives broad quantitative loss ‒ long COVID exhibits a distinct, relatively preserved Detection with disproportionate Identification impairment, calling for tailored pathways.

### Limitations of the study

One limitation of our study is that VAS and SNOT-22 scores were not systematically collected for the CRSwNP group, as these measures were not the primary focus of our analysis. Comparisons between the long COVID-19 and CRSwNP groups were performed exclusively using the BAST-24, aimed at characterizing differences in olfactory dysfunction profiles. However, it is expected that both VAS and SNOT-22 scores would be elevated in CRSwNP as well, as suggested by other authors.^24^

Another limitation arises from the subjective timing of assessment in both conditions. In the case of CRSwNP, results may vary depending on disease progression and treatment status, whereas in long COVID-19 patients, potential changes over time ‒ given the prolonged evolution of the disorder ‒ may affect the results. Longitudinal studies are needed to better assess these dynamics.

A third limitation is that although the SNOT-22 questionnaire provided valuable information on overall sinonasal-related quality of life and allowed comparisons with other rhinological conditions, it was not specifically designed to assess the impact of olfactory dysfunction. Therefore, the use of more targeted instruments, such as the Questionnaire of Olfactory Disorders (QOD), might have offered a more precise evaluation of the functional and psychosocial burden associated with smell loss in long COVID patients.

## Conclusions

Patients with long COVID-19 report persistent olfactory dysfunction with a significant impact on quality of life. However, this subjective impairment does not fully correspond to global quantitative measures of olfactory function but rather reflects a predominant deficit in odor identification. Understanding the specific characteristics of olfactory dysfunction in these patients, which differ from those observed in other rhinological disorders, can help guide more effective diagnosis, treatment and follow-up.

## ORCID ID

Aina Sansa: 0000-0003-4731-1722

Alda Cardesín: 0000-0003-1822-7599

Mariana Campos: 0000-0001-8870-5416

Carlota Rovira: 0000-0002-4357-138

Josep de Haro: 0000-0002-5940-7775

Elios Yuste: 0009-0009-7421-0389

Miguel Caballero-Borrego: 0000-0002-6105-3750

## CRediT authorship contribution statement

All the authors have made substantial contributions following the rules of the International Committee of Medical Journal Editors (ICMJE).

Design: AC. Acquisition of data: MC; CR; EY. Analysis: JH. Writing and drafting: AS. Critical Revision: MCB.

## Consent for publication

Not applicable (the manuscript does not contain data from any individual person).

## Ethics approval and consent to participate

This study was approved by the Ethics Committee of Hospital Parc Taulí (reference: 2022/5011). All procedures were conducted following the ethical standards of the institutional and national research committee and the tenets of the 1964 Helsinki Declaration and its later amendments. As this was a retrospective study, patients’ clinical data were collected without any interference with their treatment and with no risk to the patients’ physiology. The requirement for informed consent was waived and the collected data were protected from disclosure.

## Financial disclosure

The authors report no financial support or interests.

## Data availability statement

The datasets generated and/or analyzed during the current study are not publicly available due to our institutional protocols, but they will be made available from the corresponding author on reasonable request.

## Declaration of competing interest

The authors declare no have conflicts of interest.
